# Partial Unweighting in Obese Persons Enhances Tactile Transmission From the Periphery to Cortical Areas: Impact on Postural Adjustments

**DOI:** 10.3389/fnhum.2022.782028

**Published:** 2022-06-14

**Authors:** Marie Fabre, Patrick Sainton, Chloé Sutter, Laurence Mouchnino, Pascale Chavet

**Affiliations:** ^1^Aix-Marseille Université, CNRS, Laboratoire de Neurosciences Cognitives, FR 3C, Marseille, France; ^2^Aix-Marseille Université, CNRS, Institut des Sciences du Mouvement, Marseille, France; ^3^Institut Universitaire de France, Paris, France

**Keywords:** plantar sole afferents, obesity, balance, unweighting, EEG, somatosensory evoked potentials (SEP)

## Abstract

Tactile plantar information is known to play an important role in balance maintenance and to contribute to the setting of anticipatory postural adjustments (APAs) prior to stepping. Previous studies have suggested that somatosensory processes do not function optimally for obese individuals due to the increased pressure of the plantar sole resulting in balance issues. Here, we investigated whether decreasing the compression of the mechanoreceptors by unweighting the plantar sole would enhance tactile sensory processes leading to an increased stability and an accurate setting of the APAs in obese individuals. More specifically, we tested the hypothesis that the somatosensory cortex response to electric stimulation (SEP) of the plantar sole in standing obese persons will be greater with reduced body weight than with their effective weight. The level of unweighting was calculated for each participant to correspond to a healthy body mass index. We showed an increase SEP amplitude in the unweighted condition compared to the effective body weight for all participants. This increase can be explained by the reduction of weight itself but also by the modified distribution of the pressure exerted onto the foot sole. Indeed, in the unweighted condition, the vertical ground reaction forces are evenly distributed over the surface of the foot. This suggests that decreasing and equalizing the pressure applied on the plantar mechanoreceptors results in an increase in somatosensory transmission and sensory processes for obese persons when unweighted. These sensory processes are crucial prior to step initiation and for setting the anticipatory postural adjustments (i.e., thrust). These cortical changes could have contributed to the observed changes in the spatiotemporal characteristics of the thrust prior to step initiation.

## Introduction

The human foot sole is a unique structure at the interaction between our body and the support on which we are standing. The interaction between the plantar sole and the support is essential for determining body orientation in space or more particularly with respect to the support on which the feet are resting (e.g., Morasso and Schieppati, 1999; Carriot et al., 2004; Mouchnino and Blouin, 2013). Tactile receptors embedded within the skin of the foot are able to detect very small changes in force relative to the person's weight when standing motionless (e.g., Morasso and Schieppati, 1999; Fabre et al., 2021). This allows the brain to identify our balance state prior to gait initiation, for example, even though no other information from visual, vestibular, or proprioceptive origin is present (Mouchnino and Blouin, 2013). Consequently, any change in tactile plantar information from the periphery to the cortical level may alter balance control and gait initiation. For example, reducing plantar tactile information by anesthesia of the plantar sole in humans (Magnusson et al., 1990; Meyer et al., 2004) or by a section of the cutaneous nerve in cats (Bouyer and Rossignol, 2003) results in impaired postural reactions in response to a translation of the supporting surface. This is evidenced by a greater velocity of the center of pressure displacement and by a delay in the postural reaction when equilibrium is perturbed. In addition, performing a first step from a quasi-static standing requires shifting the body weight from a bipedal support to an unipedal support. This shift is initiated by the generation of propulsive forces (i.e., thrust), which in turn accelerate the center of mass (CoM) forward and toward the new base of support delineated by the supporting foot. As a rule, the higher the amplitude of the thrust, the faster the body weight transfers toward the supporting side (Mouchnino et al., 1992). Previous studies have shown that the efficacy of planning the CoM displacement is dependent on somatosensory information (Inglis et al., 1994; Timmann and Horak, 2001; Mouchnino and Blouin, 2013). For example, evidence of facilitation of tactile sensory inputs at a time when foot cutaneous information is considered to be critical for setting the anticipatory postural adjustments (APA) was observed when planning a forward step (Mouchnino et al., 2015). More recently, Fabre et al. (2020) showed that this tactile facilitation disappeared in obese individuals. Even though all mechanoreceptors are activated when walking or standing, the slow adapting afferent fibers (Merkel and Ruffini fibers) are particularly suited to respond to variations in weight bearing. Indeed, they increase their firing frequency with skin stretch or in response to forces normal to the skin and also discharge during the release of the skin deformation (Johansson and Vallbo, 1983; see Macefield, 2005 for a review). While the assumption that balance control is impaired by a decreased tactile sensitivity appears undisputed, the question remains as to whether such impairments are also linked to an altered normal force to the supporting surface applied during weight bearing. Indeed, for both obese and healthy persons carrying an external weight, larger and faster postural oscillations are observed during natural quiet standing (D'Hondt et al., 2011; Teasdale et al., 2013; Lhomond et al., 2016). Similarly, Teasdale et al. (2007) reported improved postural stability in obese individuals after weight loss. Although balance instability could indicate the inability of obese individuals to produce sufficient ankle torque, the increase in postural oscillations was also observed in highly trained athletes with values of body mass index comparable to obese individuals (Handrigan et al., 2012). The great muscular lower limb strength observed in these athletes as compared to sedentary obese individuals rules out the possibility that a muscle weakness is at the origin of postural oscillations. Rather, these results support the view that altered sensory information may be the cause of balance instability. Still, the most convincing argument is probably the observation that adding a 19-kg weighted vest onto healthy individuals decreased the somatosensory transmission as evidenced by a smaller cortical response to tactile electrical stimulation set at the perceptual threshold (Lhomond et al., 2016). Overall, these results and others (Vela et al., 1998; Hills et al., 2001) showing higher plantar foot pressure under the heel and metatarsal regions were consistent with the hypothesis that an overpressure on the plantar skin could depress the tactile afferent transmission from the periphery to the cortical areas. Inversely, studies with anorexic and bulimic patients (low Body Mass Index, BMI about 15) (Fontana et al., 2009) showed greater postural oscillations compared to healthy participants. Fontana et al. (2009) interpreted the increase of center of mass excursion as the inability to produce sufficient force. However, regarding Mouchnino et al. (2017) study showing a decrease of tactile sensory transmission from the foot sole to the cortex when healthy participants were unweighted at over 60% of their body weight, we hypothesize that a decrease in the force normal to the supporting surface could mechanically alter the tactile receptor response.

Overall, studies in both increased and decreased body weight have provided behavioral and electrophysiological evidence beyond to indicate that tactile sensory processes are decreased when the pressure on the foot sole is outside a specific range. Based on the abovementioned studies, we hypothesize that the transmission of cutaneous input to the cortex is reduced in overweight individuals due to receptor adaptation during continued skin compression. Removing the skin compression by decreasing the body weight to healthy weight values should increase somatosensory transmission. To test this hypothesis, we recorded the somatosensory evoked potential (SEP) of obese individuals in response to tactile stimulation by means of an electrical stimulus set at the perceptual threshold during two tasks (quiet standing and step initiation) in two weight conditions (100% of body weight and unweighted to healthy weight values). We were specially expecting an increase of somatosensory transmission from the foot to the cortex together with an efficient body weight transfer prior to stepping forward.

## Materials and Methods

### Participants and Methods

A total of 16 obese participants (9 men and 7 women), 18 to 55 years of age (27 ±13), took part in this study. We recruited obese persons with a body mass index (BMI) ranging from 30 to 45 (mean BMI: 35 ±5, mean body mass: 109 ±19 kg, mean height: 176 ±12 cm). Exclusion criteria were the body mass superior to 180 kg, diabetes, and/or fibromyalgia pathologies. All protocols and tasks and procedures were conducted in accordance with the ethical standards set out in the Declaration of Helsinki and approved by the local committee of St-Christophe clinic. All participants gave their written informed consent.

### Apparatus

A treadmill (M310 Anti-gravity treadmill, AlterG Inc.®, Fremont, CA, USA) based on lower body positive pressure (LBPP) technique enables an individual's body weight (BW) to be varied. LBPP technology applies a consistent and substantial lifting force opposite to BW. The AlterG® treadmill comprises an airtight flexible chamber applied distally to the volunteer's iliac crest. This creates local unweighing of the lower limbs whereas the upper body graviceptors (including vestibular receptors) still experience Earth gravity (Sainton et al., 2015). The electrical signal of the differential pressure (Pression _atmospheric_ – Pression _chamber_) was recorded along with the vertical ground reaction forces obtained from 4 dynamical load cells (XA-shear beam load cell, Sentran®, Ontario, CA, USA) located under the frame of the treadmill. The ground reaction forces were summed to compute the real BW when the participant was standing motionless. Participants wore neoprene shorts and stood barefoot on the treadmill. Initially, they remained stationary, with their arms relaxed and resting on the flexible chamber. The neoprene shorts were sealed to the inflatable chamber. The compliance properties of the chamber were such that participants' body was free to move in all directions such that participants could walk and run on the treadmill unimpaired (Cutuk et al., 2006). The participants were submitted to 2 different weight conditions: unchanged body weight and unweighted corresponding to 100%, respectively, of the body weight and to an unweighting targeting a body mass index (BMI) of 25 which corresponds to a healthy value of BMI, for each participant. The mean net weight in the unweighted condition was 72.9% (± 10%) of the 100% body weight. The 2 testing conditions were counterbalanced across the participants to avoid any order effect.

For both conditions, participants were asked to perform two tasks: “Standing” and “Stepping.” Before each task, participants were requested to self-select a side-by-side foot position with their feet placed at a natural distance apart. We ensured that the feet position remained the same throughout the experiment. For the Standing task, participants were instructed to remain in a natural quiet standing position with eyes closed during 5 s. In the Stepping task, participants were instructed to step forward with the right dominant leg at an imperative auditory signal (a 100-ms tone), again keeping their eyes closed. This imperative Go signal corresponded to the last of three 1s interspaced tones. The first two tones served as warning stimuli, giving the participants a preparation time. For reasons of homogeneity, the same tones were played in the Standing task. All participants performed 50 trials of 5 s (40 Stepping and 10 Standing) randomly generated to avoid prediction of the step. A total of two of the participants failed to maintain a centered body weight position before stepping and thus were discarded from the analyses.

### Stimulating System

**Figure 1:**
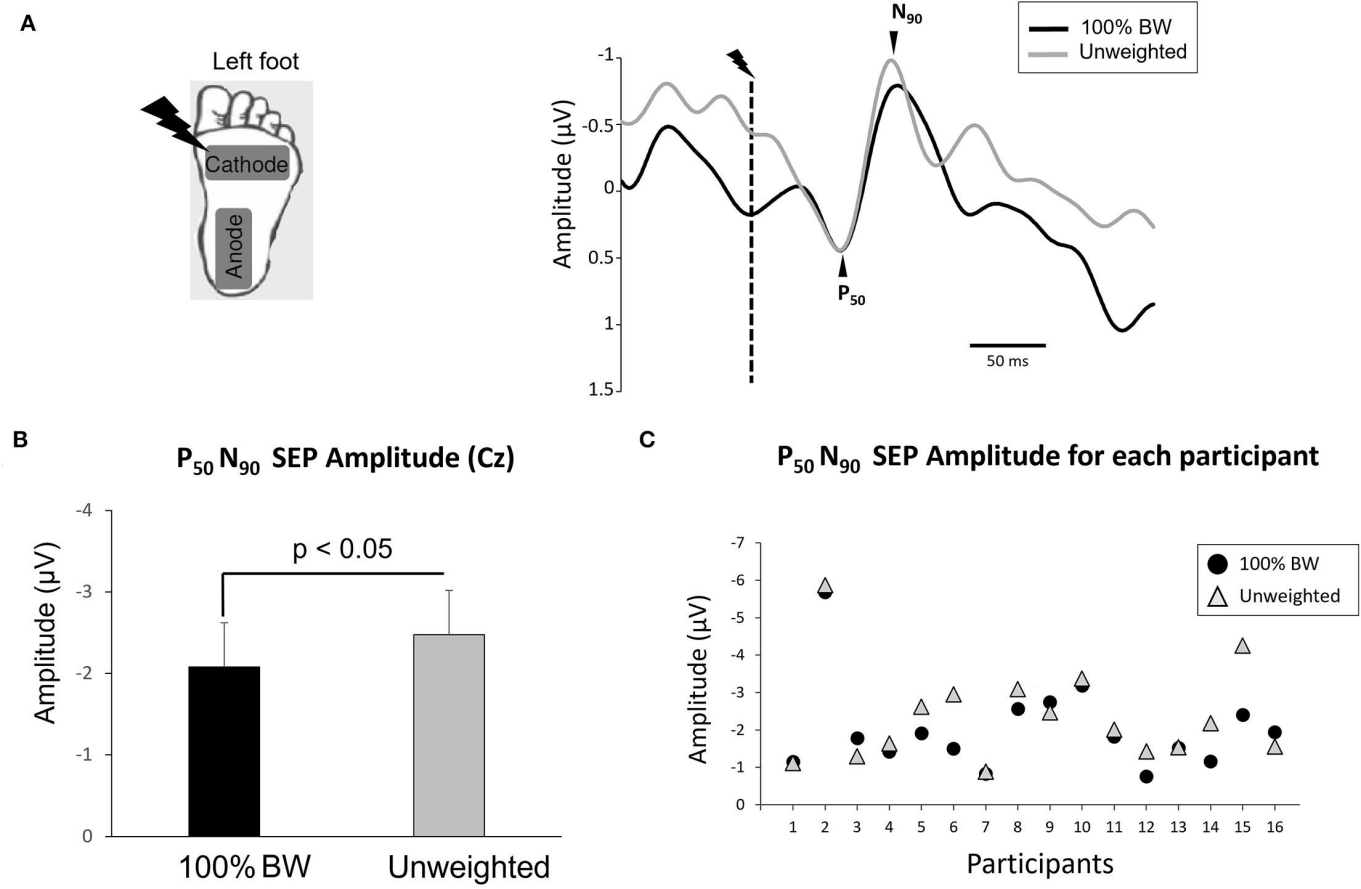
**(A)** (left panel) Position of stimulation electrodes underneath left foot. (right panel) Average of the somatosensory evoked potential for all participants over Cz electrode in 100% BW and unweighted conditions synchronized to the electric stimulation (dashed line). Note that for the figure, only we have superimposed the two conditions relative to the P50 amplitude. **(B)** Mean amplitude of the averaged P50N90 SEP evoked by the electric stimulation during 100% BW and unweighted condition for all participants (error bars are standard error across participants). **(C)** Amplitude of the averaged P50N90 SEP for each participant.

A total of four stimulations were electrically delivered to the whole plantar sole of the left foot for all participants to evoke somatosensory potential at a cortical level (Figure 1A). The reason for stimulating the left foot was 2-fold: first, all participants were right-footed and as such, the left foot is considered as the “postural” foot (Coren, 1993). Second, the crossed organization of the ascending sensory pathways could entail the analysis of cortical processes when stimulating the right for some participants and the left foot for others. Each stimulation of the foot sole was separated by 500 ms to avoid the “interference phenomenon” corresponding to the decreased potentials evoked by a stimulation when preceded by another stimulation within a 300-ms time-window (Burke and Gandevia, 1988; Morita et al., 1998). A total of 40 stimulations were analyzed for the standing task. For reasons of homogeneity, the same number of stimulations was delivered in the Stepping task but not analyzed here as this has already been reported in a previous study (Fabre et al., 2020). The stimulus was delivered by an isolated bipolar constant current stimulator (DS5 Digitimer, Welwyn Garden City, UK); the cathode was located under the metatarsal region and the anode underneath the heel (5 × 9 cm electrodes, Platinium Foam Electrodes, Figure 1A). Considering the signature of the cutaneous reflexes reported in Sayenko et al. (2009) study and the fact that repetitive pulses elicited muscle reflexes (Zehr et al., 2014), we carefully selected both the position of the electrodes to stimulate the plantar sole as a whole without targeting a specific portion of the foot and the amplitude and duration of the stimulation to avoid cutaneous reflexes (see Mouchnino et al., 2015). The stimulation consisted of a single rectangular 10-ms pulse. For each participant, the current used to stimulate the plantar sole skin was set 25% above the perceptual threshold (mean perceptual threshold: 7.2 ±1.7 mA) but remained below the threshold for evoking motor movements. Before the recording session, a forced-choice adaptive method (Ehrenstein and Ehrenstein, 1999) was used to determine the perceptual threshold of the stimulation while participants stood on the treadmill with eyes closed at their effective body weight (100%BW).

### Electrophysiological Recordings and Analyses (Standing Task)

Electroencephalographic (EEG) activity was continuously recorded from 64 Ag/AgCl surface electrodes embedded in an elastic cap (BioSemi ActiveTwo system: BioSemi, Netherlands). Specific to the BioSemi system, “ground” electrodes were replaced by Common Mode Sense active and Driven Right Leg passive electrodes. The signal was pre-amplified at the electrode sites and post-amplified with DC amplifiers, filtered online with a 0.16 Hz high pass filter (Actiview acquisition program) and digitized at a sampling rate of 1,024 Hz. Off-line filtering with a 50 Hz digital notch filter and a 0.1–35 Hz band-pass digital filter (48 dB/octave) was implemented within the BrainVision Analyzer 2 software (Brain Products, Germany). SEPs were obtained by averaging, for each participant and condition in the standing task, all synchronized epochs relative to the electrical stimulus. The average amplitude of the 50-ms pre-stimulus epoch served as baseline. We measured the SEPs over the Cz electrode as this electrode overlays the sensorimotor cortices, and on the homunculus, the feet are located on the inner surface of the longitudinal fissure. The earliest discernible positive (P50) and negative (N90) peaks following each stimulus were identified. Such peak latencies are comparable to latencies observed by Altenmüller et al. (1995) and Duysens et al. (1990) evoked by stimulating the sural nerve. The fact that the sural nerve is a primarily cutaneous nerve (Burke et al., 1981) provides an argument for the P50N90 originating from cutaneous input. The amplitude of the P50N90 waveform was measured peak-to-peak (Figure 1A). The amplitude of the SEP is known to be representative of the stimulus intensity (Salinas et al., 2000; Lin et al., 2003). Therefore, we expected the SEP amplitude to be larger in the unweighted condition as compared to the 100% BW of obese participants.

### Behavioral Recordings and Analyses

Head acceleration was recorded at a frequency of 1,000 Hz using a triaxial accelerometer (4630 Model: Measurement Specialties, USA) placed on the participants' chin. We analyzed the amplitude of head acceleration signal as an index for whole body stability supposing the whole body to act as a rigid segment (inverted pendulum model) about the subtalar joint of the feet (MacKinnon and Winter, 1993). For each trial, after applying a 4th-order low-pass Butterworth filter with 10 Hz cutoff frequency on the raw data over time, de-biasing and rectifying the signal, we computed the mean acceleration during a 3-s time-window (i.e., 1 to 4 s) which encompassed the 4 stimulation periods including the P50N90 component during the standing task.

We analyzed the vertical force distribution over the feet surface of the standing participant from the 4 dynamical strain gauges associated with the treadmill frame (two gauges were located at the front of the treadmill and two at the back). We ensured that the position of the feet remained similar throughout the experimental session, although once the flexible chamber is in place, and the participant is zipped into the AlterG treadmill, the position of the feet was predetermined. In addition, we ensured that the participants were centered by reducing the difference between the ground vertical forces of the right and left side to zero. The gauge responses were summed two-by-two to have a representation of the net front (right and left front gauges responses), rear (right and left rear gauges responses), left (front and rear left gauges responses), and right (front and rear right gauges responses) ground reaction forces. After applying a 4th-order low-pass Butterworth filter with a 10 Hz cutoff frequency on the raw data, each direction corresponding to data was normalized related to the body mass index (BMI) of each participant. For the standing task, we computed the mean force during a 3-s time-window (i.e., 1 to 4 s), which encompassed the 4 stimulation periods including the P50N90 component. To analyze the body weight transfer toward the supporting leg for the stepping task, we analyzed the left and right ground reaction forces separately. The amplitude and duration were computed between the onset and the maximal value obtained when the force stopped to increase or decrease monotonically.

### Statistical Analysis

The amplitudes and latencies of the ground reaction forces and head acceleration were submitted to repeated-measures analysis of variance (ANOVA) with weight conditions (100% BW vs. unweighted) and force localization (right, left, front, and rear) as a within-subject factors. Prior tests (Kolmogorov–Smirnov) confirmed the normality of the data. Head accelerations were also submitted to a *t*-test of means vs. a reference value set at the vestibular perceptual threshold. Due to abnormal distribution, EEG variables were submitted to a Wilcoxon test. To analyze the relation between P50N90 amplitude and the percentage of unweighting, we used the Pearson's correlation. The Tukey's *post hoc* test was used for comparisons. The level of significance was set at *p* < 0.05 for all analyses. To assess the effect size of the *t*-test and Wilcoxon test, we used Cohen's d, and for the ANOVA effect size, we used partial eta-squared.

## Results

### Somatosensory Evoked Potentials (Standing Task)

As expected, we showed a significant increase of the P50N90 SEP amplitude in the unweighted condition (*z* = 2.016, *p* = 0.044, *d* = 0.30) (Figures 1B,C). The mean P50N90 amplitude in 100%BW was 2.08 ± 0.8 μV and in the unweighted condition 2.4 ± 0.9 μV. No significant effect of unweighting on P50 latency was observed (*z* = 0.25, *p* = 0.8, *d* = 0.23; overall mean 59 ± 12 ms). Similarly, the latency of N90 was not significantly affected by the unweighted condition (*z* = 0.5, *p* = 0.6, *d* = 0.32; overall mean 95 ± 15 ms). Interestingly, the difference in SEP amplitude between 100% BW and the unweighted conditions was not correlated with the level of unweighting (i.e., percentage of body weight) to obtain a healthy weight (Pearson's correlation coefficient, *r* = 0.17) for all participants.

### Behavior

#### Ground Reaction Force

**Figure 2:**
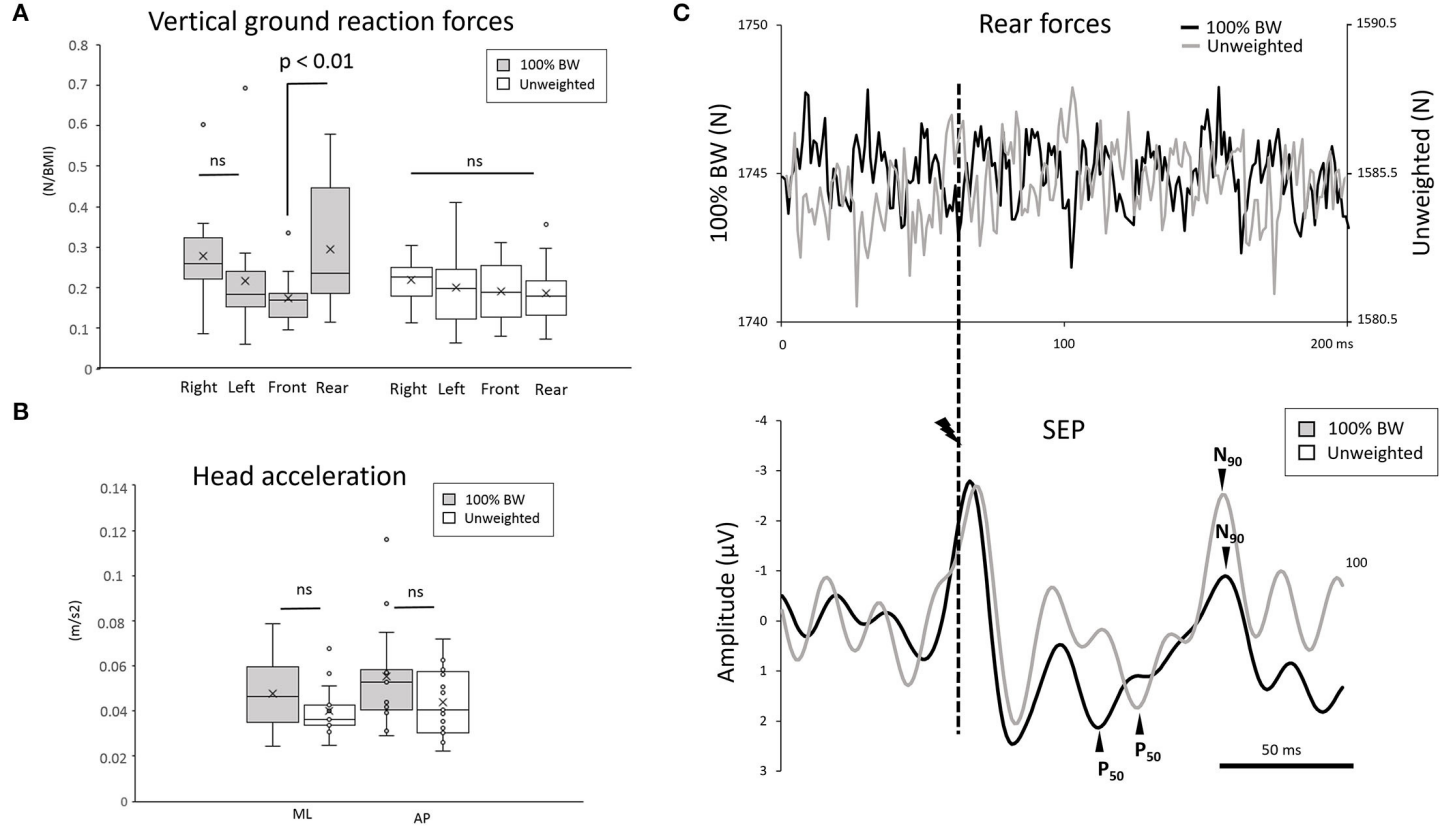
**(A)** Whisker plot of the mean vertical ground reaction force during 100% BW and unweighted conditions in the 2 directions (mediolateral and anteroposterior) for all participants (error bars are standard error across participants) during a 3s period encompassing the whole stimulation in the standing task. **(B)** Whisker plot of the mean head acceleration during 100% BW and unweighted conditions in the 4 directions (forward, backward, right, and left) for all participants (error bars are standard error across participants) during a 3-s period encompassing the whole stimulation in the standing task. **(C)** Average of the rear forces and of the somatosensory evoked potential (SEP) in the 100% BW and unweighted conditions for a representative participant. The SEP was synchronized to the electric stimulation (dashed line). The deflection observed in the SEP curve at the moment of the stimulation corresponds to the electrical stimulation artifact.

To compare changes in the variation of forces in space excluding weight reduction, we normalized the force parameters (amplitude and duration) with respect to the participants' BMI (Figures 1A,B). Interestingly, for the standing task, the ANOVA showed an interaction condition ^*^ force localization (F_3,42_ =3.72; *p* = 0.018, $\eta_{\text{P}}^{2}$ = 0.20) on the amplitude of the vertical ground reaction forces (Figure 2A). Neither the 100% BW condition (*p* = 0.38) nor the unweighted condition (*p* = 0.98) showed any change in the distribution of the forces along the lateral axis. Interestingly, in the 100% BW, the vertical force was greater over the rear than the front (*p* = 0.003) (Figure 2A). In the unweighted condition, the same force was measured in all four sectors, suggesting that the vertical forces were equally distributed on the whole support surface as no difference was observed between the rear and the front (*p* = 0.99). It should be noted that the difference for the rear force (100% BW minus unweighted conditions) was not correlated with the increase SEP amplitude (100% BW minus unweighted conditions) (Pearson's correlation coefficient, *r* = 0.34) for all participants (refer to Figure 2C, for example, for one participant).

**Figure 3:**
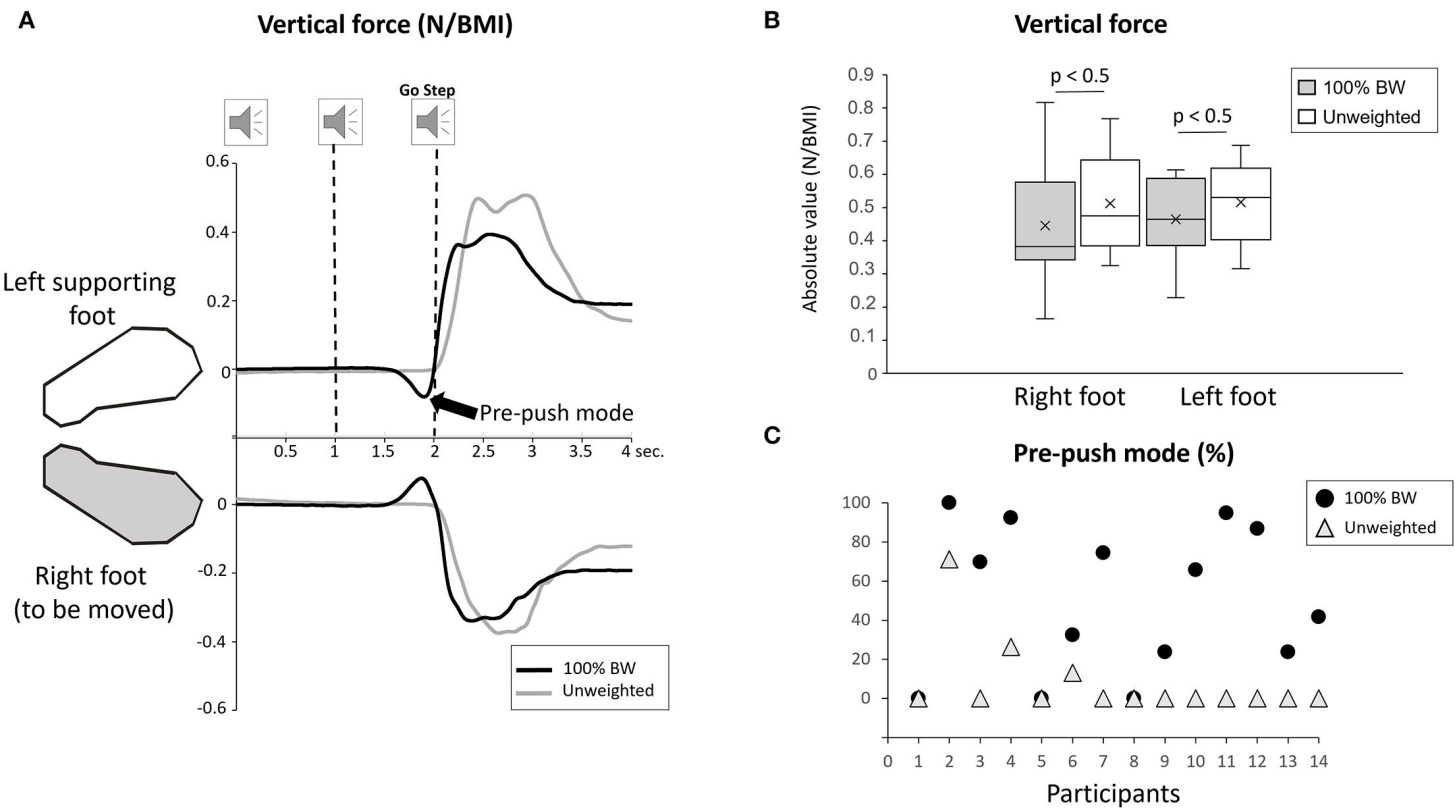
**(A)** Mean vertical ground reaction force during 100% BW and unweighted conditions under the right and the left feet for one participant in the stepping task. The participant is stepping with the right foot moving forwards. This figure shows the loading/unloading mechanism. The increase signifies a loading of the weight on the supporting foot, and the decrease represents a simultaneous unloading of the foot to be moved. Note in black the pre-push mode of body weight transfer. Speakers represent the onset of the 3-sound signals with 1-s interval. **(B)** Whisker plot of the mean amplitude (in absolute value) of the left and right vertical forces during 100% BW and unweighted conditions for all participants (error bars are standard deviation across participants). **(C)** Percentage of the occurrence of the pre-push mode for each participant in 100% BW and unweighted conditions.

Variables related to motor execution (Stepping task) were analyzed to verify whether the body weight transfer (i.e., amplitude and duration) varied across the conditions. We were particularly interested in the load/unload mechanism (Winter, 1995) during which the vertical pressure exerted under the loading left foot increases while the pressure exerted under the unloading right foot simultaneously decreases (Figure 3A). A significant condition effect was observed in the amplitude of both left and right vertical forces (F_1,13_ = 7.12; *p* = 0.019, $\eta_{\text{P}}^{2}$ = 0.34) (Figure 3B) and on their duration (F_1,13_ = 59.17; *p* < 0.001, $\eta_{\text{P}}^{2}$ = 0.71). The vertical forces were greater and lasted longer in the unweighted condition than in 100% BW. Of particular importance is the rate of change of the force (i.e., change in amplitude/duration) which is markedly smaller in the unweighted relative to 100% BW condition (F_1,13_ = 5.62; *p* = 0.033, $\eta_{\text{P}}^{2}$ = 0.20). This result suggests a smoother body weight shift (in the sense of less jerk) toward the supporting side when unweighted.

Interestingly, during the analysis of the vertical forces, two modes of body weight shift were identified. The first mode showed the load/unload mechanism and has been described above, and a second mode (which referred to the pre-pushing mode defined by Mouchnino et al., 1998) was observed in the stepping trials in 100% BW condition (Figure 3A). The force increase under the supporting foot was preceded by an early pressure shift in the opposite direction exerted under the leg destined to be the swing leg (Figure 3A). We calculated the percentage of its occurrence (Figure 3C). This pre-pushing mode was observed in 50% of the trials in 100% BW and decreased to <8% in the unweighted condition.

#### Head Acceleration

The results did not show a main condition effect (F_1,15_ = 1.75; *p* = 0.2) or a main direction effect (AP vs. ML) (F_1,15_ = 1.75; *p* = 0.2) on the mean amplitude of the head acceleration (Figure 2B). Note that the overall mean acceleration (0.047 ± 0.01 m.s^−2^) did not differ (*p* > 0.05) from the vestibular threshold (i.e., 0.048 m/s^2^; Gianna et al., 1996), suggesting that the SEP resulted mainly from tactile stimulation rather than from vestibular inputs.

## Discussion

This study was designed to gain insight into the relationship between body weight and the somatosensory transmission from the periphery to the cortical level. We used a protocol in which standing obese participants can be unweighted until a healthy effective weight was reached. Our results suggest that the unweighted condition enhanced the sensory processes hosted in the primary somatosensory (S1) cortex in obese individuals as evidenced by the larger P50N90 amplitude relative to the 100% body weight condition. Indeed, the P50N90 component was known to refer, at least partly, to signal transmission (Nelson et al., 2004; Chapman and Meftah, 2005; Bolton et al., 2011). For example, Romo and Merchant (1996) showed that neurons of S1 are the first to respond to the stimulus generated by the contact forces between the finger and the surface with a latency of 26 ms for the neurons of area 1 and 59 ms for the neurons of area 2. This neuronal discharge occurred either when the monkey actively scanned the surface or when the same stimuli were delivered passively. This suggests that the discharge is related to the tactile information and not to the movement. The latency of this neuronal discharge and the P50 latency recorded in this study suggests an enhanced activity of S1 in the unweighted condition. The relationship between the SEP and foot tactile information is also supported by Lhomond et al. (2016) study showing a reduced SEP when healthy individuals were standing while wearing a 19-kg vest (reaching a BMI of 30). Alternatively, when healthy individuals (BMI of 24; Mouchnino et al., 2017) were unweighted by up to 60 % BW, a marked decrease of the SEP was observed. Overall, these different observations are consistent with the interdependence between the flow of tactile inputs reaching the cortical areas and the level of skin compression. This idea is supported by the physiology of the mechanoreceptors (responding to pressure, especially Merkel, SA I, and Ruffini receptors, SA II, Dargahi and Najarian, 2004; for review, Vallbo and Johansson, 1984). These sensors fire when the stimulus reaches a minimum threshold and reduce the firing to even become silent when a saturation level is attained (Khalsa et al., 1996; Ge and Khalsa, 2002). For example, using isolated skin-nerve preparation (Ge and Khalsa, 2002) showed on rat limbs that the mean neuronal response for all SAIs was significantly correlated with a compressive pressure applied onto the skin: the larger the pressure, the more neuronal firing required to reach saturation (i.e., around 10 kPa of compression) where there was no significant increase in neural response or even a decreased neural response. The large compressive forces applied on the foot sole in obese individuals may have decreased the neuronal response of the mechanoreceptors. Reducing this force by about 30% of their total weight (i.e., unweighted condition) could have reactivated the receptors. Although the electrical stimulation of a class of fast-conducting myelinated peripheral nerve fibers (i.e., A-beta fibers) was identical in both conditions, it arrived at a time when the mechanoreceptors were being more activated due to the decrease of the compressive forces (unweighted condition). Consequently, this superposition might have resulted in the recruitment of additional afferent fibers, causing augmentation of the overall response observed at a cortical level (SEP).

Another salient result is the change in the spatial distribution of the foot sole pressure in obese individuals when standing motionless. While an abnormal peak pressure distribution within the foot sole in obese individuals has been previously observed (e.g., Vela et al., 1998; Hills et al., 2001; Birtane and Tuna, 2004), our results show that releasing the skin compression by unweighting the obese individuals resulted in a more even pressure over the whole foot sole. This more widely spread pressure may have also contributed to increased tactile sensory transmission in obese individuals. By contrast, a sustained force within a small area (i.e., increase vertical forces over the rearfoot in obese individuals) may increase the skin compression leading to receptor adaptation. In addition, this abnormal distribution may render non-functional, a large part of the mechanoreceptors located under the forefoot. This echoes recent findings (Strzalkowski et al., 2018) which demonstrated local variation in the distribution of cutaneous afferents across the foot sole, contrary to an even distribution previously reported (Kennedy and Inglis, 2002). Notably, they showed a higher proportion of cutaneous afferents innervating the toes as well as the lateral metatarsals and lateral arch, than that expected with an even distribution. In this regard, the unweighting that evens the pressure may optimize the use of all tactile receptors and contribute to the observed increased SEP.

Some limitations for the standing task should be considered. Even though the transmission of cutaneous input is depressed in obese individuals, head accelerations were not greater in 100% BW as compared to unweighted condition. The lack of change in postural stability (as indicated by head acceleration) may be due to the short duration of the task (i.e., 5 s). Indeed, Duarte and Zatsiorsky (1999) showed that a recording above 30 min was necessary to reveal modulation of the postural sway in terms of shift, fidget, or drift.

Unlike in the standing task, the postural behavior was markedly altered in the stepping task. The restoration of sensory flow and pressure distribution over the plantar sole with unweighing modified both the sensory and biomechanical context of the step initiation. The unweighting condition revealed an unexpected smoother body weight shift toward the supporting side (i.e., reduced jerk) even though a greater vertical force amplitude was observed. A total of two explanations that cannot be yet disentangled can be envisaged. The increase vertical force on the supporting foot may be the result of the reactivation of sensory inputs that inform the body about an equilibrium state (Fabre et al., 2021). This could have contributed to changing the setting of the APAs (i.e., increasing the weight bearing on the supporting foot). However, to verify this possibility, future research is needed. Tri-dimensional recording of pelvis acceleration may provide answers about the center of mass excursion since the pelvis segment, closer to the feet, may reflect more discernable modulation of the postural sway (Winter, 1995). Changes in the weight shift would rather represent a functional response. Indeed, the pre-push mode observed in obese individuals in half of the stepping movement (100% BW) could set the body in motion facilitating the forthcoming body weight shift toward the supporting side. Contributing to the body weight transfer as was observed in below-knee amputees, the pre-pushing mode could be considered as APA (Mouchnino et al., 1998). Another interpretation is that the early shift may be involved in evaluating the support conditions to plan the body weight transfer toward the supporting foot. Indeed, similar shifts in the opposite direction were observed in the cat after a lesion of the sensory-motor cortex, which provoked sensory deficits in the limb contralateral to the lesion (Birjukova et al., 1989). Decreasing and equalizing vertical reaction forces in the unweighted condition may decrease the need of the pre-push mode. Indeed, this reduction was reported for 11 obese participants out of 14.

In conclusion, we have provided neurophysiological evidence that unweighting the lower limbs results locally in an increased tactile transmission from the foot to the cortex for obese individuals. This suggests that the pressure applied onto the foot skin of obese individual is too large and damages the functioning of the receptors. Based on the current results and other studies, a critical range of pressure applied on the foot skin, where mechanoreceptors are embedded, is needed to enhance for enhancing tactile sensory transmission. Moreover, we have shown that artificially reducing the body weight of obese volunteers affects the dynamics of anticipatory postural adjustments which are known to be predominantly centrally programmed.

## Data Availability Statement

The raw data supporting the conclusions of this article will be made available by the authors, without undue reservation.

## Ethics Statement

The studies involving human participants were reviewed and approved by Aix-Marseille Université. The patients/participants provided their written informed consent to participate in this study.

## Author Contributions

MF, LM, PS, and PC contributed to the conception and design of the work. MF, LM, PC, and CS contributed to the acquisition, analysis, or interpretation of data for the work and contributed to the writing of the work or revising it critically. All authors contributed to the article and approved the submitted version.

## Conflict of Interest

The authors declare that the research was conducted in the absence of any commercial or financial relationships that could be construed as a potential conflict of interest.

## Publisher's Note

All claims expressed in this article are solely those of the authors and do not necessarily represent those of their affiliated organizations, or those of the publisher, the editors and the reviewers. Any product that may be evaluated in this article, or claim that may be made by its manufacturer, is not guaranteed or endorsed by the publisher.
